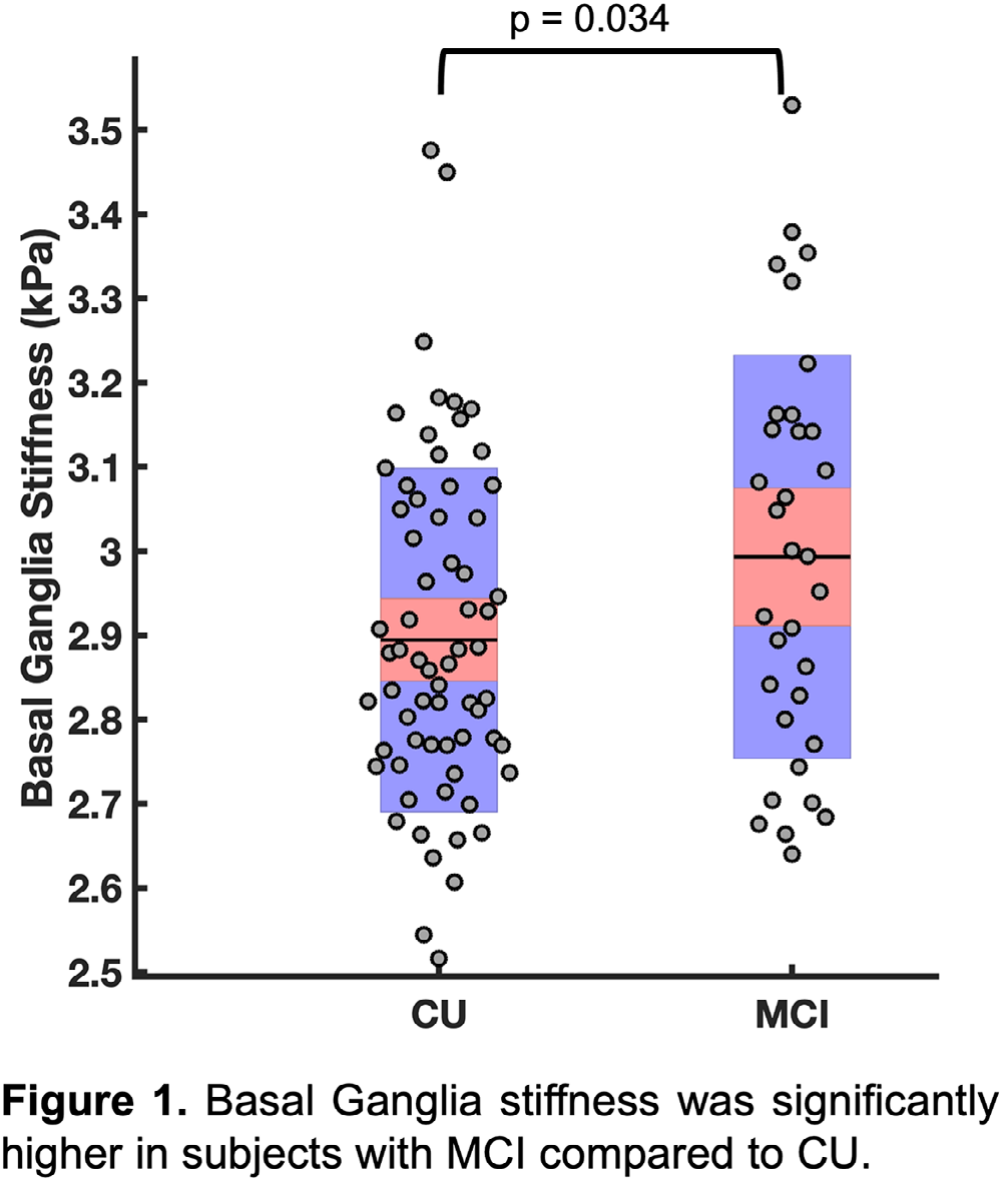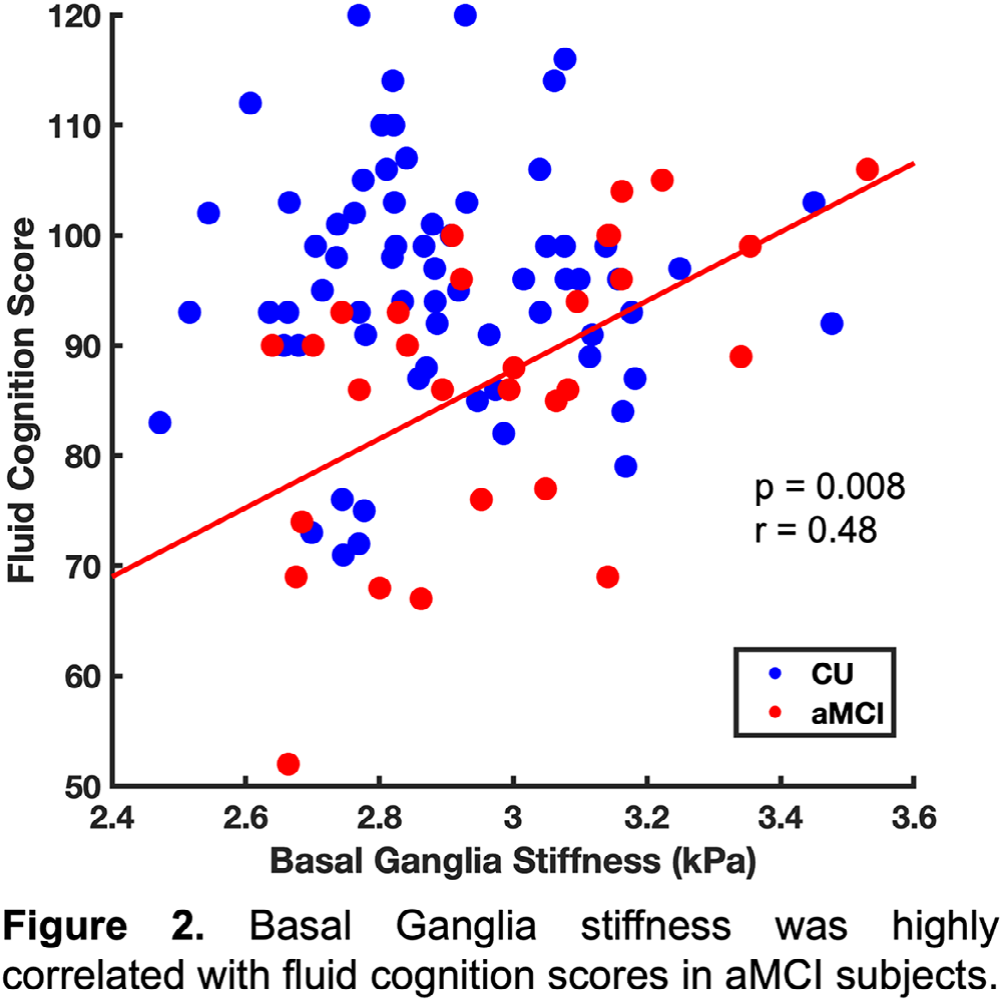# Basal Ganglia Mechanical Stiffness is Associated with Fluid Intelligence in Amnestic Mild Cognitive Impairment

**DOI:** 10.1002/alz.083610

**Published:** 2025-01-09

**Authors:** Mary K Kramer, Peyton L Delgorio, Alexa M Diano, Grace McIlvain, Kyra E Twohy, Olivia M Bailey, Matthew L. Cohen, Alyssa M Lanzi, Christopher R. Martens, Curtis L Johnson

**Affiliations:** ^1^ University of Delaware, Newark, DE USA

## Abstract

**Background:**

Magnetic resonance elastography (MRE) is an MRI technique that uses mild, externally applied vibrations to quantify the mechanical properties of tissues in vivo. MRE measures, such as stiffness, have been shown to be sensitive to changes in brain health with aging and neurodegeneration. Here we used MRE to characterize differences in brain mechanical properties between individuals with amnestic mild cognitive impairment (aMCI) and cognitively unimpaired subjects (CU).

**Method:**

A cohort of 67 cognitively unimpaired subjects (21M/46F; 60‐82y) and a cohort of 34 subjects with aMCI (11M/23F; 60‐89y) completed an MRE scan to assess their brain mechanical properties. From this, we quantified basal ganglia (BG) stiffness in each subject including the caudate, pallidum, putamen, and nucleus accumbens. Subjects also completed the NIH toolbox cognition battery from which we examined fluid cognition composite score, which reflects logic and reasoning skills.

**Result:**

We found a significant group difference in stiffness of the basal ganglia (BG). Interestingly, aMCI subjects had significantly higher BG stiffness than CUs (2.99 vs. 2.89 kPa, p<0.05; Figure 1). Within the aMCI group, BG stiffness was positively correlated with fluid cognitive score (r=0.48, p<0.01; Figure 2), where higher scores were associated with greater stiffness; the same relationship in the CU group was not statistically significant.

**Conclusion:**

Higher BG stiffness found in the aMCI group may reflect aspects of aMCI pathology and progression that MRE can sensitively detect. Most MRE studies report that healthy brains are associated with higher stiffness than those with neurodegenerative pathology, with our previous work showing that aMCI participants had softer hippocampi than age‐matched CUs (Delgorio, 2023). Our findings in the BG are surprising in this context but may indicate a compensatory mechanism that occurs during early progression of aMCI and results in increased stiffness, which was previously suggested by Murphy (2016). Here we also provide the first evidence that higher BG stiffness in aMCI is associated with better cognitive function, which further points to higher stiffness being compensatory rather than pathological. The neurobiological basis of this compensatory increase in BG stiffness of aMCI subjects requires further study but could be influenced by acute neuroinflammatory processes.